# HOX transcription factors are potential targets and markers in malignant mesothelioma

**DOI:** 10.1186/s12885-016-2106-7

**Published:** 2016-02-11

**Authors:** Richard Morgan, Guy Simpson, Sophie Gray, Cheryl Gillett, Zsuzsanna Tabi, James Spicer, Kevin J. Harrington, Hardev S. Pandha

**Affiliations:** Institute of Cancer Therapeutics, Faculty of Life Sciences, University of Bradford, Richmond Road, Bradford, BD7 1DP UK; Faculty of Health and Medical Sciences, University of Surrey, Guildford, UK; Division of Cancer Studies, King’s College London, Guy’s Hospital, London, UK; Institute of Cancer and Genetics, University of Cardiff School of Medicine, Cardiff, UK; Targeted Therapy Team, Chester Beatty Laboratories, The Institute of Cancer Research, London, UK

**Keywords:** Mesothelioma, *HOX* genes, HXR9, HOXB4, Overall survival

## Abstract

**Background:**

The *HOX* genes are a family of homeodomain-containing transcription factors that determine cellular identity during development and which are dys-regulated in some cancers. In this study we examined the expression and oncogenic function of *HOX* genes in mesothelioma, a cancer arising from the pleura or peritoneum which is associated with exposure to asbestos.

**Methods:**

We tested the sensitivity of the mesothelioma-derived lines MSTO-211H, NCI-H28, NCI-H2052, and NCI-H226 to HXR9, a peptide antagonist of HOX protein binding to its PBX co-factor. Apoptosis was measured using a FACS-based assay with Annexin, and *HOX* gene expression profiles were established using RT-QPCR on RNA extracted from cell lines and primary mesotheliomas. The in vivo efficacy of HXR9 was tested in a mouse MSTO-211H flank tumor xenograft model.

**Results:**

We show that *HOX* genes are significantly dysregulated in malignant mesothelioma. Targeting *HOX* genes with HXR9 caused apoptotic cell death in all of the mesothelioma-derived cell lines, and prevented the growth of mesothelioma tumors in a mouse xenograft model. Furthermore, the sensitivity of these lines to HXR9 correlated with the relative expression of *HOX* genes that have either an oncogenic or tumor suppressive function in cancer. The analysis of *HOX* expression in primary mesothelioma tumors indicated that these cells could also be sensitive to the disruption of HOX activity by HXR9, and that the expression of *HOXB4* is strongly associated with overall survival.

**Conclusion:**

*HOX* genes are a potential therapeutic target in mesothelioma, and *HOXB4* expression correlates with overall survival.

## Background

The *HOX* genes are a family of transcription factors characterized by highly conserved DNA- and co-factor binding domains. This conservation has been driven by their roles in some of the most fundamental patterning events that underlie early development [1]. Most notable of these is the patterning of the anterior to posterior axis, for which a precise spatial and temporal order in the expression of *HOX* genes is required. This is achieved in part through a chromosomal arrangement whereby *HOX* genes are present in closely linked clusters allowing the sharing of common enhancer regions. In mammals there are four such clusters (A–D), containing a total of 39 *HOX* genes [1]. The relative position of each *HOX* gene 3′ to 5′ within the cluster is reflected in a number of key attributes, including the spatial and temporal order of expression, whereby the 3′ most genes are expressed earlier than their 5′ neighbors. The nomenclature of the *HOX* genes reflects this precise chromosomal ordering, with members of each cluster being numbered with respect to the 3′ end, thus for example, the 3′ most member of cluster B is *HOXB1* [2].

The 3′ to 5′ order of *HOX* genes is reflected not only in their expression patterns but also in their DNA binding specificities and co-factor interactions. For example, the products of the 3′ *HOX* genes (1 to 9) bind to another transcription factor, PBX, which modifies their binding specificity to DNA [3], influences their nucleocytoplasmic distribution [3], and also determines whether a HOX protein will activate of repress transcription of downstream target genes [4]. This interaction with PBX is mediated through a highly conserved hexapeptide region on HOX proteins 1–9 that binds to a cleft in PBX [3, 5]. Once PBX has bound it can recruit other specific co-factors, including MEIS, which can then further modify HOX activity [6].

Although *HOX* genes were initially characterized as key developmental genes, they also function in adult stem cells to promote proliferation [7], and subsequently in their progeny to confer lineage-specific identities [8]. Furthermore, *HOX* genes are strongly dys-regulated in cancer, and generally exhibit greatly increased expression. This differential change in expression in cancer may reflect the apparent ability of some *HOX* genes to function as tumor suppressors and some as oncogenes. Thus for example, *HOXA5* acts as a tumor suppressor in breast cancer by stabilizing P53 [9], whilst forced expression of *HOXB6* can immortalize fibroblast cells [10]. Further examples of this phenomenon are listed in Table 1.

**Table 1:** *HOX* genes with potential oncogenic or tumor suppressor functions

Gene	O / S	Evidence	Reference
*HOXA1*	O	Transforms non-malignant mammary epithelial cells	[28]
*HOXA9*	O	Key oncogene in leukemia	[29]
*HOXB3*	O	Pro-survival and proliferation gene in leukemia	[29]
*HOXB4*	O	Pro-survival and proliferation gene in leukemia	[29]
*HOXB5*	O	Transfection can immortalize fibroblast cells	[21]
*HOXB6*	O	Transfection can immortalize myelomonocytic cells	[10]
*HOXB9*	O	Promotes tumorogenesis in breast cancer	[30]
*HOXC4*	O	High expression in malignant prostate cells	[31]
*HOXA4*	S	Blocks spread of ovarian cancer cells	[32]
*HOXA5*	S	Identified as a tumor suppressor gene in breast ca	[9]
*HOXC8*	S	Expression inversely related to progression	[33]
*HOXC12*	S	Promotes cell differentiation in follicular lymphoma	[22]
*HOXD12*	S	Silenced in melanoma cells	[23]

*O HOX* gene with oncogenic activity, *S HOX* gene with tumor suppressor activity

The dys-regulation of *HOX* genes has been demonstrated in a range of cancers, and in some it has been shown to be a potential therapeutic target through the use of a peptide, HXR9. HXR9 prevents PBX binding to HOX and triggers apoptosis in malignant cells, whilst sparing normal adult cells [11–17]. Although these studies include non-small cell lung cancer (NSCLC) [16], they do not encompass mesothelioma, a malignancy of the mesothelium cells which is most frequently found in the lung and is associated with long term exposure to asbestos [18]. Mesothelioma has limited treatment options and generally a very poor prognosis [18], and therefore finding novel therapeutic approaches in this disease is an important goal. In this study we show that *HOX* dys-regulation is present in cell lines derived from mesothelioma, and in primary tumors, usually with a significant increase in the expression of those *HOX* genes that behave as oncogenes. Furthermore, antagonism of the HOX / PBX interaction in these cell lines triggers apoptosis, with malignant cells generally being considerably more sensitive to HXR9 than cells derived from non-malignant mesothelium cells.

## Methods

### Cell lines and culture

The cell lines used in this study are listed in Table 2. They were obtained from the ATCC through LGC Standards Ltd (UK), and were cultured according to the instructions on the LGC Standards website.

**Table 2:** Mesothelioma-derived cell lines used in this study

Cell line	Source	IC50 HXR9 (μM)	Ref
Met-5a	Normal mesothelium cells from pleural fluid	98	[34]
NCI-H28	Pleural effusion	18	ATCC
MSTO-211H	Biphasic mesothelioma (fibroblast morphology)	28	[35]
NCI-H2052	Pleural effusion (epithelial morphology)	45	ATCC
NCI-H226	Squamous carcinoma; mesothelioma (epithelial morphology). This cell line was derived from non-small cell lung cancer, although it was subsequently found to have a number of mesothelioma-related properties, including the expression of mesothelin.	107	ATCC, [36]

### Synthesis of HXR9 and CXR9 peptides

HXR9 is an 18 amino acid peptide consisting of the previously identified hexapeptide sequence that can bind to PBX and nine C-terminal arginine residues (R9) that facilitate cell entry. The N-terminal and C-terminal amino bonds are in the D-isomer conformation, which has previously been shown to extend the half-life of the peptide to 12 h in human serum [14]. CXR9 is a control peptide that lacks a functional hexapeptide sequence but which includes the R9 sequence. The sequences of these peptides have been published previously [13]. All peptides were synthesized using conventional column based chemistry and purified to at least 80 % (Biosynthesis Inc., USA).

### Imaging of cell cultures

Cells were plated in 6-well plates using 2 ml of medium and allowed to recover for at least 24 h. When approximately 60 % confluent, cells were treated with the active peptide HXR9 (60 μM) or the control peptide CXR9 (60 μM) for 3 h.

### Immunohistochemistry for HOXA4, HOXA9, and HOXB4

Expression of HOXA4, HOXA9, and HOXB4 in mesothelioma and normal mesothelium tissue was investigated using 3 μm-thick, formalin fixed, paraffin embedded tissue array sections (MS081, US Biomax, Rockville, MD, USA). Immunohistochemical analysis was performed using a monoclonal rabbit anti-HOXB4 antibody (ab676093, 1:100 dilution, Abcam, Cambridge, UK), a polyclonal rabbit anti-HOXA4 antibody (ab131049, 1:500 dilution, Abcam, Cambridge, UK), and a polyclonal rabbit anti-HOXA9 antibody (ab191178, 1:75 dilution, Abcam, Cambridge, UK). The ABC detection method with peroxidase block (DakoCytomation) was used for all of these primary antibodies. Antigen retrieval was performed using pH 9.0 Tris/EDTA buffer (DakoCytomation) and heating in a microwave for 23 min.

### Analysis of cell death and apoptosis

Cells were treated with HXR9 or CXR9 as described above. Cell viability was assessed using the MTS assay (Promega) according to the manufacturer’s instructions. Cells were harvested by incubating in trypsin-EDTA (Sigma) at 37 °C until detached and dissociated. Apoptotic cells were identified using flow cytometry (Beckman Coulter Epics XL Flow) and the Annexin V-PE apoptosis detection kit (BD Pharmingen) as described by the manufacturer’s protocol. Caspase-3 activity was measured using the EnzCheck Caspase-3 Assay Kit (Molecular Probes), using the protocol defined by the manufacturer.

### RNA purification and reverse transcription

Total RNA was isolated from cells using the RNeasy Plus Mini Kit (Qiagen) by following the manufacturer’s protocol. The RNA was denatured by heating to 65 °C for 5 min. cDNA was synthesized from RNA using the Cloned AMV First Strand Synthesis Kit (Invitrogen) according to the manufacturer’s instructions.

### Quantitative PCR

Quantitative PCR was performed using the Stratagene MX3005P real-time PCR machine and the Brilliant SYBR Green QPCR Master Mix (Stratagene). The following primers were designed to facilitate the unique amplification of *β-actin*, *c-Fos*, and each *HOX* gene:

HsBeta-ActinF: 5′ ATGTACCCTGGCATTGCCGAC 3′

HsBeta-ActinR: 5′ GACTCGTCATACTCCTGCTTG 3′

HscFos1F: 5′ CCAACCTGCTGAAGGAGAAG 3′

HscFos1R: 5′ GCTGCTGATGCTCTTGACAG 3′

HsHOXA1F: 5′ CTGGCCCTGGCTACGTATAA 3′

HsHOXA1R: 5′ TCCAACTTTCCCTGTTTTGG 3′

HsHOXA4F: 5′ CCCTGGATGAAGAAGATCCA 3′

HsHOXA4R: 5′ AATTGGAGGATCGCATCTTG 3′

HsHOXA5F: 5′ CCGGAGAATGAAGTGGAAAA 3′

HsHOXA5R: 5′ ACGAGAACAGGGCTTCTTCA 3′

HsHOXA9F: 5′ AATAACCCAGCAGCCAACTG 3′

HsHOXA9R: 5′ ATTTTCATCCTGCGGTTCTG 3′

HsHOXB3F: 5′ TATGGCCTCAACCACCTTTC 3′

HsHOXB3R: 5′ AAGCCTGGGTACCACCTTCT 3′

HsHOXB4F: 5′ TCTTGGAGCTGGAGAAGGAA 3′

HsHOXB4R: 5′ GTTGGGCAACTTGTGGTCTT 3′

HsHOXB5F: 5′ AAGGCCTGGTCTGGGAGTAT 3′

HsHOXB5R: 5′ GCATCCACTCGCTCACTACA 3′

HsHOXB6F: 5′ ATTTCCTTCTGGCCCTCACT 3′

HsHOXB6R: 5′ GGAAGGTGGAGTTCACGAAA 3′

HsHOXB9F: 5′ TAATCAAAGACCCGGCTACG 3′

HsHOXB9R: 5′ CTACGGTCCCTGGTGAGGTA 3′

HsHOXC4F: 5′ CGCTCGAGGACAGCCTATAC 3′

HsHOXC4R: 5′ GCTCTGGGAGTGGTCTTCAG 3′

HsHOXC8F: 5′ CTCAGGCTACCAGCAGAACC 3′

HsHOXC8R: 5′ TTGGCGGAGGATTTACAGTC 3′

### Mice and in vivo trial

All animal experiments were conducted in accordance with the United Kingdom Coordinating Committee on Cancer Research guidelines for the Welfare of Animals in Experimental Neoplasia and were approved by the University of Surrey Research Ethics Committee. The mice were kept in positive pressure isolators in 12 h light / dark cycles and food and water were available *ad libitum*.

Athymic nude mice were inoculated subcutaneously with a suspension of 2.5 × 10^6^ MSTO-211H cells in culture media (100 μl). Once tumors reached volumes of approximately 100 mm^3^, mice were injected IP with PBS or 25 mg/Kg HXR9 in PBS (injection volume 100 μl), every 4 days. The mice were sacrificed after 36 days and the tumors were excised for RNA extraction, as previously described [12]. Each treatment group contained ten mice. The mice were monitored carefully for signs of distress, including behavioral changes and weight loss.

### Patient characteristics

Primary mesothelioma samples were obtained from 16 male and five female patients. The median patient age at diagnosis was 63.9 years (range, 38.2–79.53 years) and median survival was 9.04 months (range, 0.23–81.85 months). Recruitment was via a specialized multidisciplinary thoracic oncology clinic, involving thoracic surgeons, radiation oncologists, and medical oncologists. Histopathology and imaging review was undertaken for all patients. Patients underwent tumor resection at the Department of Thoracic Surgery, Guy’s & St Thomas’ NHS Foundation Trust. Tumor samples were confirmed as mesothelioma by pathological examination and categorized as a sarcomatoid, biphasic, or epithelial type using an antibody panel that included BerEP4, CEA, TTF1, Calretinin, WT1, CK5, MNF116, and EMA. Pseudoanonymised tissues and data were collected by the KHP Cancer Biobank, and subsequently released for this study in accordance with NHS REC approval number 07/H0804/91. Written informed consent was obtained from patients when they agreed to their tissue samples being included in the Biobank, it was not required for the specific use of these tissues in this project.

### Statistical analysis

All values are given as the mean of three independent experiments and error bars show the standard error of the mean. Categorical variables were compared using Student’s *t*-test or a one-way ANOVA. Survival curves were generated using the Kaplan-Meier method and compared using the log-rank test. A *p value* < 0.05 was considered to be significant.

## Results

### HOX gene expression in mesothelioma-derived cell lines and primary tumors

**Fig. 1:**
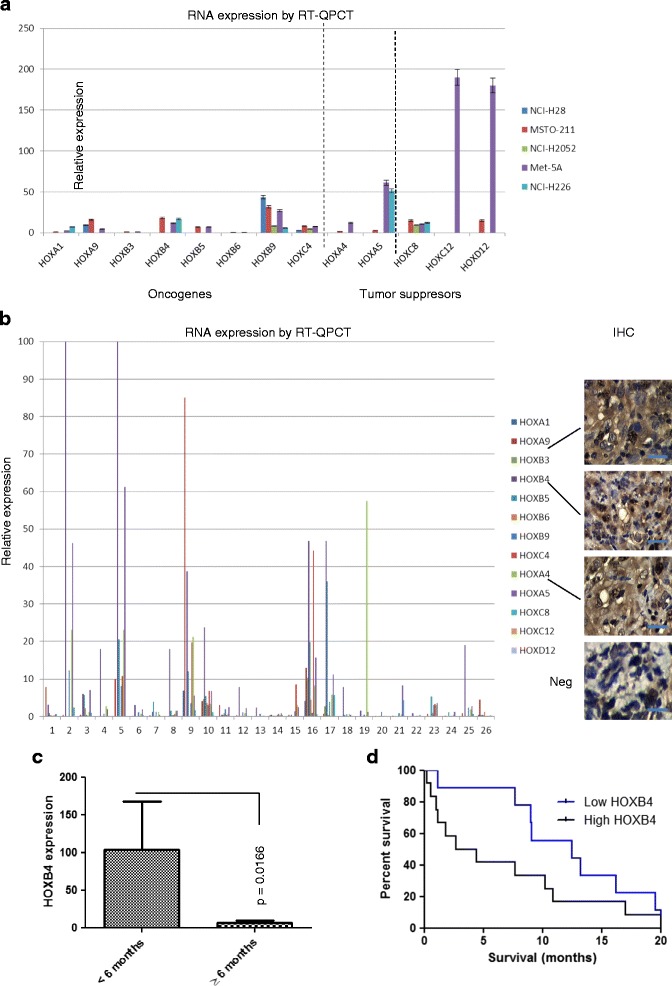
Expression of *HOX* genes in cell lines derived from mesothelioma (**a**) and (**b**) primary mesothelioma tumors. These genes were previously shown to function as either oncogenes or tumor suppressors (see Table 1 for more detail). The relative levels of RNA for each gene are shown as a ratio with *Beta-actin* (×10000 for NCI-H28, NCI-H2052 and NCI-H226, ×100 for primary mesothelioma tumors, Met-5A, and MSTO-211). For the cell lines (**a**) each value is the mean of three experiments, and error bars show the SEM. For the primary tumors (**b**) the expression of each *HOX* gene is shown for each individual tumor. The values shown are the mean of three technical repeats. No error bars are included in order to simplify the figure, although all repeats were within 10 % of the mean value. For three of the HOX genes, (HOXA4, HOXA9, and HOXB4), the protein expression was also determined using immunohistochemistry and an example of each staining from a single tumor is shown. Scale bar: 20 μm. Neg, negative – no primary antibody. **c** *HOXB4* tumor expression, as determined using quantitative real-time PCR, is significantly higher amongst patients surviving for less than 6 months after diagnosis (values on the y-axis are the ratio of *HOXB4* to *Beta-actin* expression × 10000). **d** *HOXB4* expression is associated with a shorter overall survival. Kaplan-Meier survival curves for patients with high- and low-*HOXB4* expressing tumors (*p* = 0.041). The cut-off point between high- and low-expression was determined as the midpoint between the mean values of *HOXB4* expression shown in (**c**), which was 53

In order to assess the expression of *HOX* genes in mesothelioma we used QPCR to measure RNA levels in four cell lines derived from this malignancy: NCI-H28, NCI-H2052, NCI-H226, and MSTO-211H, together with Met-5A which is derived from non-malignant mesothelium cells (Table 2). *HOX* gene expression was also studied in primary mesothelioma tumors. The expression of *HOX* genes within each cell line and between cell lines varied considerably, with MSTO-211H and Met-5A generally having far higher expression than the other cell lines. The only *HOX* genes expressed uniquely by a single cell line were *HOXC12* and *HOXD12*, in Met-5A. Analysis of *HOX* genes that are known to have oncogenic or tumor suppressive functions (Table 1) likewise reveals considerable variation, although Met-5A showed higher expression of the potential tumor suppressor genes *HOXA4* and *HOXA5* compared to the malignant cell lines (Fig. 1a). We also assessed the expression of these *HOX* genes in 21 primary tumors using RT-QPCR, as well the protein expression of the three most strongly expressed, HOXA4, HOXA9, and HOXB4 at the protein level using immunohistochemistry (Fig. 1b).

### High HOXB4 tumor expression is associated with poor overall survival

We looked for associations between the RNA expression levels of the different *HOX* genes and patient survival. The tumors of patients surviving less than 6 months had a significantly higher expression of *HOXB4* (*p* = 0.0166; Fig. 1c), and likewise a Kaplan-Meier analysis of overall survival (OS) showed that high *HOXB4* tumor expression was associated with a significantly shorter OS (*p* = 0.041; Fig. 1d).

### HXR9 is cytotoxic to mesothelioma cells

**Fig. 2:**
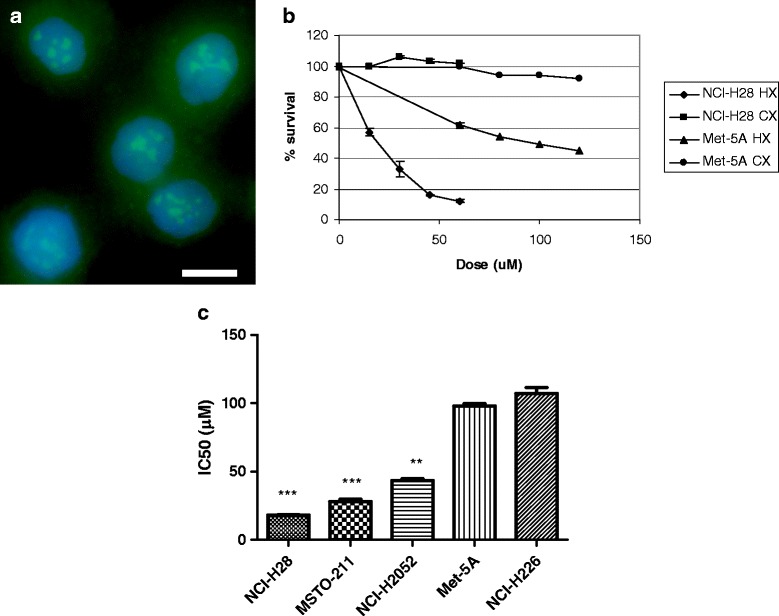
HXR9 is cytotoxic in mesothelioma-derived cell lines. **a** Fluorescent micrograph of NCI-H28 cells treated with 18 μM FITC-HXR9 (*green*) showing uptake into the nucleus and cytoplasm. Cell nuclei are stained blue. Scale bar: 5 μm. **b** Sample dose response curves for HXR9 and CXR9 treatment of NCI-H28 and Met-5A cell lines. **c** IC50 values for HXR9 in mesothelioma-derived cell lines. All incubations with HXR9 were for 2 h. Each value is the mean of five experiments, error bars show the SEM. The NCI-H28, MSTO-211H, and NCI-H2052 cells were all significantly more sensitive to killing by HXR9 than Met-5a (**, *p* < 0.01; ***, *p* < 0.001)

Given the high level of *HOX* expression in the mesothelioma cell lines, we treated cells with the HOX / PBX inhibitor HXR9 that has previously been shown to block HOX / PBX interactions and trigger apoptosis in a number of other cancers [11–17]. Use of a fluorescently labeled version of HXR9 demonstrated that it can be taken up by the cell lines studied here (Fig. 2a), and the MTS assay for cell viability revealed that HXR9 is cytotoxic in all five cell lines (Fig. 2b,c; Table 2). The non-malignant line Met-5A is amongst the least sensitive with an IC50 of 98 μM, whilst the NCI-H28 cell line is the most sensitive with an IC50 of 18 μM (Fig. 2c, Table 2).

### HXR9 triggers apoptosis

**Fig. 3:**
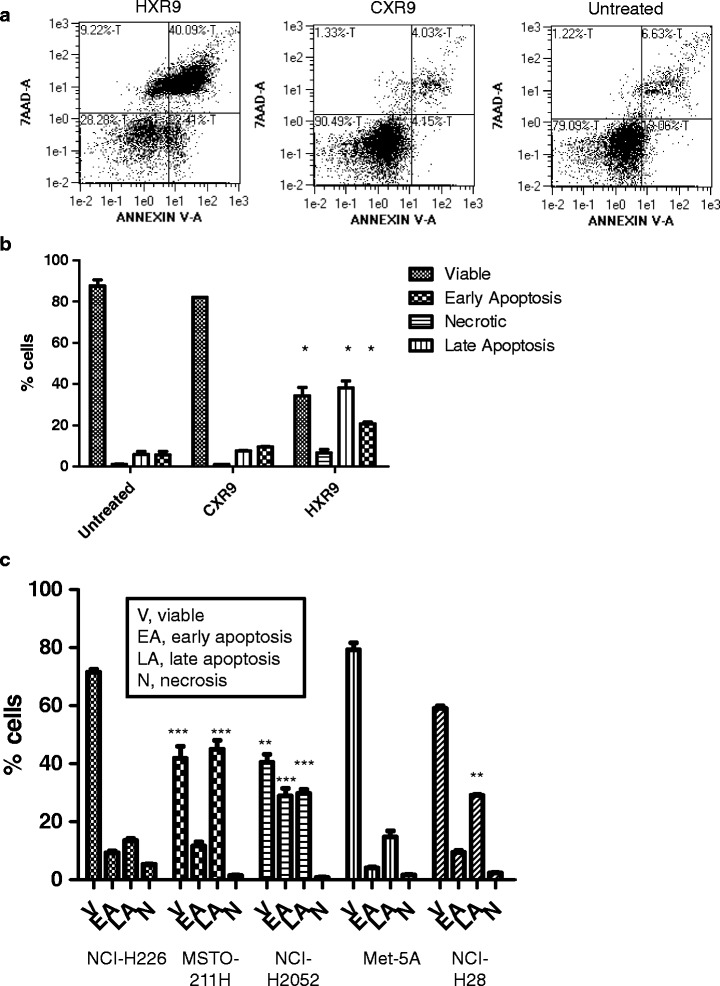
HXR9 triggers apoptosis in treated cells. The mechanism of cell death was analyzed using a FACS-based Annexin / 7AAD method to assess early and late apoptosis. **a** Sample dot plots for NCI-H28 cells treated with 18 μM HXR9 for 2 h. Viable cells sort to the lower left hand quadrant (low Annexin / 7AAD staining), whilst cells in early and late apoptosis sort to the lower and upper right hand quadrants, respectively. Necrotic cells are in the upper left hand quadrant. **b** Apoptosis in NCI-H28 cells either untreated or incubated with 18 μM HXR9 or CXR9 for 2 h. The values are the means of three experiments, error bars show the SEM. Treatment with HXR9 causes a significant increase in apoptosis (*, *p* < 0.05). **c** Summary of apoptosis data for all five cell lines. V – viable cells, EA – cells in early apoptosis, LA – cells in late apoptosis, N-necrotic cells. The values are the means of three experiments, error bars show the SEM. ***, *p* < 0.001; **, *p* < 0.01 relative to the corresponding values for Met-5a

Previous studies have suggested that the mechanism of cell death when HOX function is blocked by HXR9 is primarily through apoptosis [11–17]. To establish whether this is also the case of the mesothelioma derived cell lines, a standard FACS based assay for apoptosis-associated cell membrane changes was used. This involves the use of Annexin V that binds to membrane components usually located on the cytoplasmic side but which relocate to the external surface during apoptosis [19], and a fluorescent dye (7AAD) which binds to DNA but can only enter cells when membrane integrity has been lost. This assay revealed that all the mesothelioma cell lines underwent apoptosis when treated with HXR9 at the relevant IC50 (Fig. 3), with the non-malignant cell line Met-5A showing the lowest level of apoptosis and NCI-H2052 the highest (Fig. 3c).

**Fig. 4:**
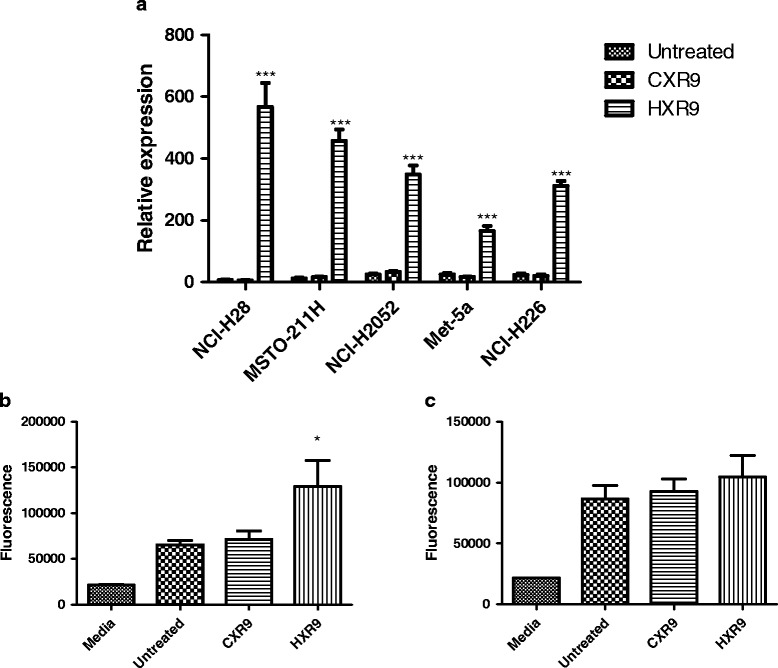
Mechanisms of cell death. **a** Induction of *cFos* in mesothelioma-derived cell lines. The amount of *cFos* RNA was determined by QPCR in cells either untreated or treated with HXR9 or CXR9 for 2 h at the IC50 for each. Expression is shown relative to *Beta-actin* (×10000). The values are the means of three experiments, error bars show the SEM. *** indicates a *p* < 0.001 compared to *cFos* expression in untreated cells. **b** Caspase 3 activation in NCI-H28 cells and Met-5A cells (**c**). The values are the means of three experiments, error bars show the SEM. * indicates a *p* < 0.05 compared to caspase 3 activity in untreated cells

The induction of apoptosis by HXR9 is thought to depend, at least in part, upon a rapid increase in *cFos* expression [14], and QPCR analysis of the HXR9 treated cells correspondingly showed a significant increase in *cFos* in all of the cell lines, with the smallest increase in Met-5A and the largest increase in the most sensitive cell line, NCI-H28 (Fig. 4a). Correspondingly NCI-H28 also showed the greatest increase in Caspase 3 activity (a protease involved in the apoptotic pathway; Fig. 4b), whilst Met-5A failed to show any significant increase in caspase activity (Fig. 4c).

### Sensitivity to HXR9 correlates with the expression of specific HOX genes

**Fig. 5:**
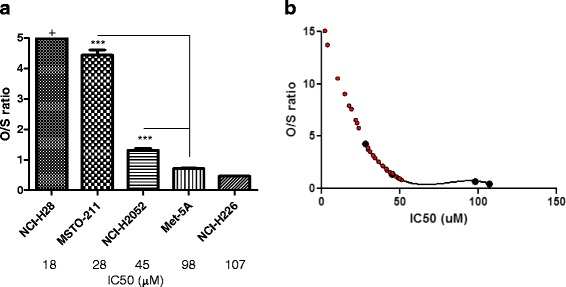
**a** Ratios of oncogenic to tumor suppressor *HOX* gene expression (O/S ratio) in mesothelioma derived cell lines. The values are the means of three experiments, error bars show the SEM. *** denotes *p* < 0.001 compared to the O/S ratio in the non-malignant mesothelium cell line Met-5A; (+) denotes that no expression of tumor suppressor *HOX* genes was detected in NCI-H28 so the ratio could not be calculated. **b** Correlation between sensitivity to HXR9 and O/S ratio. The IC50 for killing by HXR9 is plotted against the O/S ratio for each cell line (*black dots*), revealing a possible negative correlation between the two. This relationship can be modeled using a third order polynomial equation (r^2^ = 1), which is shown as a solid black line. The O/S ratio of each primary tumor was used to calculate its predicted sensitivity to HXR9 (*red dots*)

The expression of *HOX* genes with previously identified oncogenic or tumor suppressor properties (Table 1; Fig. 1), raises the possibility that the expression profile of these genes could determine the sensitivity of cells to HXR9. To assess this we divided *HOX* genes into two groups – those with potential oncogenic functions, and those with possible tumor suppressor functions. An expression ratio was obtained by dividing the total expression of genes in the former group with that in the latter (‘O/S ratio’). This revealed that the most sensitive cell line, NCI-H28, has the highest O/S ratio, whilst Met-5a and the least sensitive malignant line, NCI-H226, have the lowest O/S ratios (Fig. 5a). Plotting these ratios against the IC50 for each cell line suggest a positive correlation between the O/S ratio and sensitivity (Fig. 5b). Furthermore, the calculated O/S ratios for the primary mesothelium tumors indicate that these cells could also be sensitive HXR9 (Fig. 5b).

### HXR9 blocks the growth of mesothelioma tumors in vivo

**Fig. 6:**
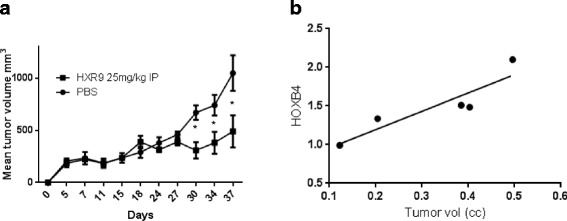
HXR9 blocks the growth of mesothelioma tumors in vivo. **a** The growth of MSTO-211H tumors in xenograft mice injected IP every 4 days with PBS or 25 mg/Kg HXR9, for a total of five times. P values were calculated using a Student’s *t*-test for each time point, “*” indicates statistical significance (*p* = 0.008, *p* = 0.037, and *p* = 0.041 for days 30, 34, and 37, respectively. **b** *HOXB4* expression in the excised tumors from PBS-treated mice, as determined by QRT-PCR. There was a linear relationship between tumor size and *HOXB4* expression (r^2^ = 0.8278; *p* = 0.0321)

In order to determine whether HXR9 could also block tumor growth in vivo, we established a xenograft mouse flank model using the MSTO-211H cell line. Mice were injected IP with either PBS or 25 mg/Kg HXR9 in PBS every 4 days after tumors had grown to a mean volume of 100 mm^3^. HXR9 significantly retarded tumor growth compared to PBS alone (Fig. 6a). In tumors from mice injected with PBS only, we found a significant, linear relationship between the expression of *HOXB4* and final tumor size (r^2^ = 0.8278; *p* = 0.0321; Fig. 6b).

## Discussion

The dys-regulation of *HOX* genes in cancer is now well established, and in many cases a putative function for individual *HOX* genes has been established [20]. Despite a high degree of sequence and regulatory conservation between *HOX* genes, there is apparently a wide range of cancer specific functions which include both oncogenic and tumor suppressing activities. Thus for example the fifth gene of the *HOXA* complex, *HOXA5*, acts primarily as a tumor suppressor in breast cancer through stabilizing p53 [9], whilst its closely related counterpart in the *HOXB* cluster, *HOXB5*, can be defined as an oncogene as it can immortalize fibroblast cells upon transfection [21].

None of these studies have as yet addressed whether *HOX* genes are dys-regulated in mesothelioma, but here we show that cell lines derived from mesothelioma as well as primary mesothelioma cells have distinctly different *HOX* expression patterns from the Met-5a cell line that is derived from normal mesothelium. One of the most striking differences is the expression of *HOXC12* and *HOXD12* by Met-5a but not by any of the mesothelioma cell lines. *HOXC12* is repressed in follicular lymphoma through hypermethylation of its promoter, and has also been implicated in the differentiation of follicle cells [22], both of which suggest a possible function in tumor suppression. Likewise, the function of *HOXD12* has not been defined, but it has been shown to be silenced in melanoma cells through the methylation of its promoter [23].

Another oncogenic *HOX* gene that we found to be up-regulated in primary mesothelioma tumors was *HOXB4*. High *HOXB4* expression levels were associated with shorter OS, suggesting that *HOXB4* expression is a potential prognostic factor in this malignancy. We also found that there was a positive, linear relationship between *HOXB4* expression and tumor growth in a mouse model of human mesothelioma. Given the functional redundancy amongst HOX proteins, this finding that *HOXB4* was the only *HOX* gene among the 39-strong family to have any prognostic significance seems unexpected. However, there are a number of other cancers for which a single *HOX* gene alone acts as a prognostic marker, and the identity of the *HOX* gene in each case varies from one malignancy to another. Examples include *HOXC6* in gastric cancer, *HOXB8* in ovarian cancer, and *HOXD3* in breast cancer [24]. This might reflect the embryonic origins of different cancer types, as *HOX* gene expression in adult cells tends to reflect their developmental origin [25]. From a practical view point, there are currently no reliable markers of OS in mesothelioma [26], and the use of *HOXB4* as a prognostic marker in this context therefore justifies further evaluation.

In this study we have found that the ratio of expression between *HOX* genes with a putative oncogenic function and those that have tumor suppressor activity (‘O/S ratio’) predicts which mesothelioma cell lines are most sensitive to HXR9, a peptide that prevents HOX proteins binding to PBX and has been shown to cause apoptosis in other malignancies [11–17]. The O/S ratio may indicate the degree to which malignant cells are dependent on the activity of oncogenic *HOX* genes for their proliferation and survival, a concept similar to the idea of ‘oncogene addiction’ [27], which would explain their sensitivity to HXR9. The extent to which this is true is yet to be determined, but at a more practical level the O/S ratio might act as a biomarker for the sensitivity of mesothelioma cells to HXR9, and could ultimately be used to select patients that might benefit from this therapeutic approach.

## Conclusion

Our findings indicate that the *HOX* genes are widely dysregulated and often strongly upregulated in mesothelioma, and that elevated *HOXB4* expression predicts shorter OS in mesothelioma patients. Targeting the interaction between *HOX* proteins and their PBX cofactor causes apoptosis in mesothelioma cells in vitro and retards tumor growth in vivo, indicating that *HOX* proteins are a potential therapeutic target in this malignancy.

## Abbreviations

O/S ratio: ratio of oncogenic to tumor suppressor *HOX* gene expression
OS: overall survival
